# Exploration of Microbial Diversity and Community Structure of Lonar Lake: The Only Hypersaline Meteorite Crater Lake within Basalt Rock

**DOI:** 10.3389/fmicb.2015.01553

**Published:** 2016-01-22

**Authors:** Dhiraj Paul, Shreyas V. Kumbhare, Snehit S. Mhatre, Somak P. Chowdhury, Sudarshan A. Shetty, Nachiket P. Marathe, Shrikant Bhute, Yogesh S. Shouche

**Affiliations:** Microbial Culture Collection, National Centre for Cell Science, Savitribai Phule University of Pune CampusPune, India

**Keywords:** 16S rRNA gene, microbial diversity, soda lake, basalt rock, Lonar Lake, India

## Abstract

Lonar Lake is a hypersaline and hyperalkaline soda lake and the only meteorite impact crater in the world situated in basalt rocks. Although culture-dependent studies have been reported, a comprehensive understanding of microbial community composition and structure in Lonar Lake remains elusive. In the present study, microbial community structure associated with Lonar Lake sediment and water samples was investigated using high-throughput sequencing. Microbial diversity analysis revealed the existence of diverse, yet largely consistent communities. *Proteobacteria* (30%), *Actinobacteria* (24%), *Firmicutes* (11%), and *Cyanobacteria* (5%) predominated in the sequencing survey, whereas *Bacteroidetes* (1.12%), *BD1-5* (0.5%), *Nitrospirae* (0.41%), and *Verrucomicrobia* (0.28%) were detected in relatively minor abundances in the Lonar Lake ecosystem. Within the *Proteobacteria* phylum, the *Gammaproteobacteria* represented the most abundantly detected class (21–47%) within sediment samples, but only a minor population in the water samples. *Proteobacteria* and *Firmicutes* were found at significantly higher abundance (*p* ≥ 0.05) in sediment samples, whereas members of *Actinobacteria*, Candidate division TM7 and *Cyanobacteria* (*p* ≥ 0.05) were significantly abundant in water samples. Compared to the microbial communities of other hypersaline soda lakes, those of Lonar Lake formed a distinct cluster, suggesting a different microbial community composition and structure. Here we report for the first time, the difference in composition of indigenous microbial communities between the sediment and water samples of Lonar Lake. An improved census of microbial community structure in this Lake ecosystem provides a foundation for exploring microbial biogeochemical cycling and microbial function in hypersaline lake environments.

## Introduction

Naturally occurring alkaline water bodies are unique and important ecosystems that offer valuable insights into how microbial communities cope with extreme growth conditions. High concentrations of carbonate and its complex salts, scarcity of Mg^2+^ and Ca^2+^, high salinity, especially due to NaCl, and a high pH (9.0–12.0) are the hallmarks of these lakes (Wani et al., 2006; Antony et al., 2010; Sorokin et al., 2015). To date, a limited number of tropical lakes have been explored, especially those in the East African Rift Valley including Lake Nakuru and Lake Bogoria (Duckworth et al., 1996; Jones and Grant, 1999; Chandrasekhar et al., 2002; Vargas et al., 2004; Wani et al., 2006). Many of these alkaline lakes also have high productivity rates (Melack and Kilham, 1974; Grant, 2006) and contain microbial ecosystems that are active in nutrient and carbon cycling. However, a unified picture of microbial populations in these lakes has yet to emerge.

Lonar Lake is a tropical soda lake formed nearly 50,000 years ago due to a meteorite impact. It is the only example of an impact crater formed on volcanic flood basaltic rocks, making it geologically comparable to craters on the Martian surface (Fudali et al., 1980; Hagerty and Newsom, 2003). The lake is also a sink to a number of fresh water springs and streams with no discernible outlets (Joshi et al., 2008). Environmental constraints have selected for a distinctive microbial community; methylotrophs, purple- sulfur and non-sulfur photosynthetic bacteria, and other unusual bacteria such as *Nitritalea halalkaliphila, Indibacter alkaliphilus, Cecembia lonarensis*, and *Georgenia satyanarayanai* were isolated and reported from this lake (Jones et al., 1998; Zavarzin et al., 1999; Antony et al., 2010; Surakasi et al., 2010; Kumar et al., 2012; Srinivas et al., 2012; Sultanpuram et al., 2015). Microbial life in sediments of this lake was previously examined using clone libraries and Denaturant Gradient Gel Electrophoresis, revealing the presence of the bacterial phyla *Firmicutes, Proteobacteria*, and *Actinobacteria* (Wani et al., 2006; Surakasi et al., 2010). Since these technologies offer only limited resolution, the phylogenetic profile of this lake's microbial communities in water column and sediment remains rudimentary and only offers limited insights into associated microbial processes. The aim of this study is to obtain a detailed view of the bacterial diversity and community structure in Lonar Lake water and sediment using a high-throughput 16S rRNA gene sequencing approach, to improve our understanding of autochthonous community composition within this complex and extreme lake ecosystem.

## Materials and methods

### Site description and sampling

Three sediment samples (S1, S2, and S3) from 40 to 50 cm depth and three corresponding water samples (W1, W2, and W3) from 10 to 20 cm depth used in this study were collected from three different locations (19°98.222′N, 76°50.698′E; 19°97.435′N, 76°50.342′E; 19°97.532′N, 76°51.337′E; respectively) of the Lonar Lake located in Buldhana district, Maharashtra, India during October 2013. The sediment samples from each site were collected using sterile scoopers in sterile polyethylene bags, while water samples were collected directly into sterile bottles. The surface temperature of the water was determined (27°C ± 0.5) on site. The pH values measured were within the range of 9.5–10.0. For the microbiological investigation, samples were stored immediately in ice and transported to the laboratory within 24 h and stored at 4°C. Genomic DNA from the respective sediment and water samples was extracted within 72 h after sample collection and stored at −20°C. For chemical analysis, water samples were collected following the standard procedure that include filtering them through 0.45 μm filter membrane for removing the debris particles and acidification with trace element grade HNO_3_ (pH 2.0), while the un-acidified filtered samples were collected for the analysis of major anions (Mukherjee et al., 2008).

### Analysis of chemical parameters

Major anions (${\text{SO}}_{4}^{2-}$, ${\text{NO}}_{3}^{-}$, ${\text{CO}}_{3}^{-}$, and Cl^−^) of the samples were measured with an ion chromatograph (Dionex, USA). Total Fe, Mg, Co, Ni, B, and Cu were determined by atomic absorbance spectrometer (AAnalystTM 200, ParkinElmer, USA). Levels of Na, Ca, and K were quantified by using flame photometer (52A Flame Photometer Perkin–Elmer, USA). Total organic carbon (TOC), total nitrogen (TN), total dissolved solids (TDS), total phosphorus (TP), and NH_3_ were analyzed using standard methods (APHA, 1998).

### DNA extraction from samples

Total DNA was extracted from all water and sediment samples using PowerWater DNA Extraction Kit (MoBio Laboratories, Inc., USA) and PowerSoil DNA Extraction Kit (MoBio Laboratories, Inc., USA), respectively. Briefly for water DNA extraction, 250 mL water was filtered through 0.22 μm membrane filter and the filter paper was used for subsequent DNA extraction following manufacturer's instruction. Prior to sediment DNA extraction, all sediment samples were homogenized under aseptic conditions, and 250 mg sediment was used for total DNA extraction using a PowerSoil DNA Kit (MoBio, Carlsbad, CA), following manufacturer's instructions. Yield and quality of the extracted DNA samples were checked on 0.8% agarose gel, and DNA concentration was measured using a NanoDrop ND-1000 spectrophotometer (Nano Drop technologies, Willingminton, USA). All the extracted DNA samples were stored at −20°C until further processing.

### Preparation of 16S rRNA gene amplicon libraries and sequencing

For amplicon library preparation, the universal primer set of 341F (5′-CCTACGGGAGGCAGCAG-3′) and 518R (5′-ATTACCGCGGCTGCTGG-3′) specific for the V3 variable region of 16S rRNA gene were used (Bartram et al., 2011). The forward primer included Ion adaptor “A” and barcode sequences and the reverse primer was attached to Ion adapter “p1” sequence. Details of the primers used are presented in Table S1. Each 50 μL PCR reaction contained: 20 ng of template DNA, 25 μL AmpliTaq Gold 360 Master Mix (Life Technologies, Invitrogen division, Darmstadt, Germany) and 1.0 μL of 10 mM each primer. The PCR amplification was performed with an initial denaturation step at 94°C for 4 min; followed by 35 cycles of denaturation at 94°C for 30 s, annealing at 56°C for 30 s, elongation at 72°C for 30 s and a final extension at 72°C for 10 min. Post-amplification, amplicons were purified using 1.5X Agencourt AMPure XP Beads (Beckman Coulter, Inc., Brea, CA) following manufacturer's protocol. Prior to sequencing, all the amplicon products were assessed for fragment size distribution and DNA concentration using a Bioanalyzer 2100 (Agilent Technologies, USA). Further, 10 pM each amplified DNA fragments were attached to the surface of Ion Sphere Particles (ISPs) using an Ion Xpress Template 200 Kit (Life Technologies, USA) and were clonally amplified through emulsion PCR, following the manufacturer's instructions. Sequencing of the amplicon libraries was carried out on an Ion Torrent PGM system (Life Technologies, USA) using 316 chip. After sequencing, all the individual sequence reads were filtered within the PGM software to remove low-quality reads, 3′ adaptor regions and polyclonal sequences.

### Ion torrent data analysis

After Ion Torrent PGM sequencing, the raw data was obtained in Fastq format. The pre-processing of the raw sequences, i.e., Fastq to Fasta file (sequence) conversion, qual (quality score), and data trimming were done by using MOTHUR version 1.34.3 (http://www.mothur.org/wiki) (Schloss et al., 2009) with the following conditions of minimum length: 130 bp, maximum length: 150 bp, maximum homopolymer: 5, maximum ambiguity: 0 and average quality score: 20. The trimmed good quality sequences were merged into a single FASTA file for further analysis in QIIME v1.7 (Quantitative Insights Into Microbial Ecology) (http://qiime.org/) (Caporaso et al., 2010). The sequences were aligned to bacterial 16S rRNA gene sequence, and clustering or OTU picking was performed by using a reference-based OTU picking approach with Silva database (Silva_111_release, March 2015) (Pruesse et al., 2007). The OTU picking was carried out using UCLUST method with a similarity threshold of 97% (Edgar, 2010). Taxonomic assignments were performed using RDP naïve Bayesian classifier (version rdp_classifier_2.10.1, released 29.10.2014) (Wang et al., 2007). Using QIIME, alpha diversity indices namely Chao1, ACE, Shannon, Simpsons' index, and Goods coverage were calculated. The sequences obtained from Ion Torrent sequencing are available at the NIH Sequence Read Archive (SRA) under the project accession number SRP059522.

### Statistical analyses

Experimental observations were recorded with three replications (*n* = 3) and data were expressed as mean ±SD. Two-tailed paired *t*-tests were used for statistical analysis. Comparative analysis was performed to determine statistically significant structural differences within the sediment and water samples of Lonar Lake by two-sided Fisher exact tests using Statistical Analysis of Metagenomic Profiles (STAMP) program (Parks and Beiko, 2010). The co -occurrence and -exclusion of the bacterial groups in the sediment and water samples was performed using CoNet analysis (V.0.6) by Cytoscape (V-3.02) (Shannon et al., 2003). Five statistical measures were used for co-occurrence network analysis: Spearman and Pearson's measures of correlation and Hellinger distance, Kullback-Leibler dissimilarity, and Bray-Curtis dissimilarity measures. Statistical significance was assigned by measure specific and edge specific permutation and bootstrap scoring with 1000 iterations. The *p*-value was calculated by the z-score distribution where high *p*-value signified co-exclusion and low *p*-value signified co-presence of the bacterial group in a particular habitat (Faust et al., 2012). The *p*-values retrieved from multiple statistical methods were merged by using Simes method. For multiple test correction, Benjamini-Hochberg correction was used (*p* ≥ 0.05). To obtain link among the connecting nodes, a minimum support of two statistical methods was used (Faust et al., 2012). The relationship among the samples with respect to family level distribution was ascertained using Unweighted Pair Group Method with Arithmetic Mean (UPGMA). In order to correlate bacterial community composition within the test samples and those previously reported from other soda lakes [(viz., Deep Lake (Colorado); Lake Chaka (China); Soap Lake (Washington); Organic Lake (Eastern Antarctica); Mono Lake (California); and Echo Lake (Colorado)], two UPGMA clustering was performed (details of all other samples used for this analysis is presented in Table S2). MVSP 3.1 software was used for UPGMA analysis (Paul et al., 2015).

## Results

### Geochemical properties of samples

Geochemical properties of both water and sediment samples were analyzed to understand the physicochemical conditions in these microbial habitats. These samples differed markedly in terms of carbon-nitrogen stoichiometry, with water samples showing a lower C:N ratio compared to the sediments. All samples exhibited characteristic geochemical properties associated with a hypersaline soda lake, particularly with respect to pH, salinity, TOC, total N, total P, ${\text{CO}}_{3}^{2-}$, and Cl^−^ content (Table 1). Additionally, geochemical analyses also revealed the presence of sulfates, ammonium and metals (such as Ni, Co, Cu, and Mg) in both sediment and water samples.

**Table 1:** **Physiochemical analysis of water and sediment samples**.

**Chemical Parameters**	**Water**	**Sediment**
Total Dissolved Solids (TDS) (g/L)	1.09±0.06	1.04±0.02
Total Organic Carbon (TOC) (g/L)	0.002±0.0003	0.25±0.05
Total Nitrogen (TN) (g/L)	0.004±0.0005	0.19±0.03
Total Phosphorus (P) as ${\text{PO}}_{4}^{3-}$ (mg/L)	0.3±0.02	3000.06±20.02
Sodium as Sodium chloride (Na) (g/L)	3.8±0.09	1.7±0.02
Carbonates (${\text{CO}}_{3}^{-}$) (g/L)	0.18±0.01	0.23±0.03
Chlorides (Cl^−^) (g/L)	0.56±0.07	0.21±0.06
Ammonia (NH_3_) (mg/L)	0.9±0.08	3±0.02
Sulfates (${\text{SO}}_{4}^{2-}$) (mg/L)	0.004±0.0006	50±3.84
Calcium as (Ca) (mg/L)	0.7±0.04	520±3.02
Cobalt as Co (mg/L)	0.03±0.001	2±0.31
Nickel (Ni) (mg/L)	0.01±0.002	0.9±0.04
Boron as B (mg/L)	0.02±0.003	1±0.2
Magnesium as Mg (mg/L)	1.7±0.5	1100±8.94
Potassium as K (mg/L)	0.5±0.03	50±1.83
Iron as Fe (mg/L)	0.09±0.001	2000±10.93
Copper as Cu (mg/L)	0.002±0.0001	5.3±0.512

*All data represent mean of triplicate (±) SD*.

### Microbial diversity analysis

Sequence datasets obtained after removal of low-quality sequences were trimmed, and only sequences of good quality were retained for subsequent analyses. A total of 11,90,896 high-quality sequences were obtained from all the samples. Taxonomic assignment of these sequences to define operational taxonomic units was based on a similarity threshold of 97%. Good's coverage (≥95%) indicated a high degree of sequence coverage (Table 2). Non-parametric indicators (ACE, Chao1, Simpson, and Shannon) were computed to evaluate community diversity characteristics of bacteria associated with hypersaline Lonar Lake sediments and the corresponding water samples. The assessment of diversity indices revealed differences in the complexity of bacterial communities in water and sediment samples from the lake, indicating the existence of a large bacterial community in Lonar Lake. Furthermore, when compared to sediment samples (S1, S2, S3), the corresponding water samples lacked both in diversity and complexity. Incidentally amongst sediment samples, S1 had the highest values of species richness indices of Chao1 (73143.25), ACE (73189.04) and also a higher Shannon diversity index (10.93), followed by samples S2 and S3 (Table 2). Overall across all the samples Shannon indices exhibited the following trend: W2<W3<W1<S3<S2<S1, confirming a higher diversity of bacterial communities in the sediment samples as compared to the water samples (Table 2).

**Table 2:** **Sequence summary and calculation of alpha diversity indices of the sediment and water samples**.

**Sample ID**	**Sample nature**	**Raw reads**	**Assigned reads**	**OTUs**	**Coverage (%)**	**Richness and diversity indices**			
						**ACE**	**Chao1**	**Shannon (H)**	**Simpsons (1/D)**
S1	Sediment	2000415	530536	515	0.98	73189.04	73143.25	10.93	0.994
S2	Sediment	996815	446224	396	0.96	53824.68	53563.58	10.23	0.989
S3	Sediment	209554	100577	383	0.95	36556.59	34211.12	10.01	0.977
W1	Water	245344	99230	119	0.97	10303.09	10349.17	8.09	0.975
W2	Water	20756	9142	62	0.95	1648.65	1654.78	6.82	0.967
W3	Water	11136	5187	93	0.95	2473.5	2247.17	7.77	0.984

### Bacterial community composition and structure

**Figure 1:**
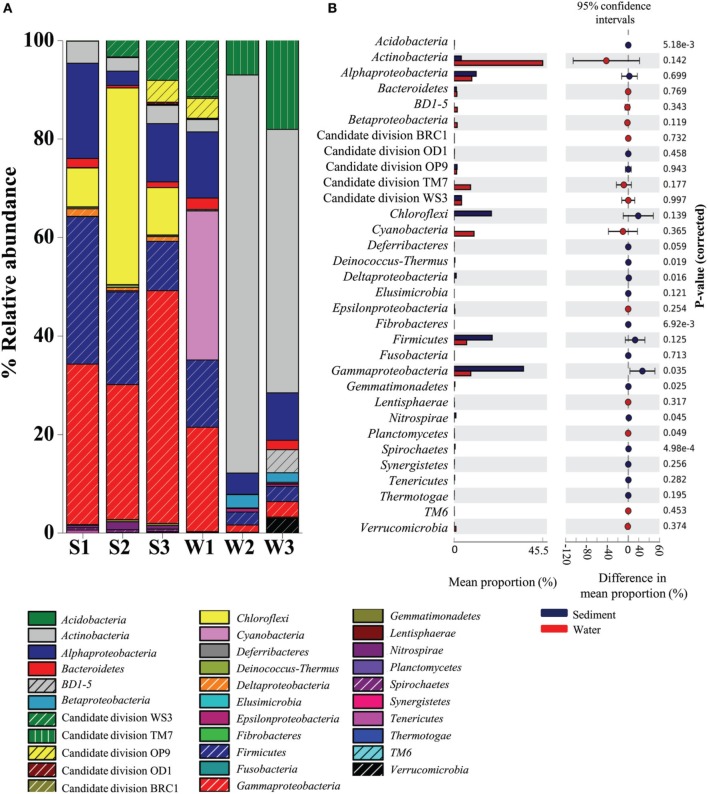
**Distribution of major phylogenetic groups of bacteria in (A) across the sediment and water samples, and (B) overall distribution between sediment and water samples of the Lonar Lake**. Bacterial abundance in the sediment sample has a positive difference between proportions (blue circles), whereas bacterial abundance in the water sample has a negative difference between proportions (red circles). Bars on the left represent the proportion of each bacterial phyla abundance in the samples. Bacterial abundance difference with a *p*-value of >0.05 were considered to be significant.

A total of 28 bacterial phyla were detected in the Lonar Lake samples. The ten most abundant phyla contributed up to 88% of the total bacterial diversity (Figure 1). Taxonomic analysis based on relative abundance revealed *Proteobacteria* (30%) to be the most predominant phyla followed by *Actinobacteria* (24%), *Firmicutes* (11%), *Cyanobacteria* (5%), and Candidate division TM7. *Bacteroidetes, BD1-5, Nitrospirae, Verrucomicrobia*, and *Spirochaetes* represented a relatively minor portion of the total diversity (Figure 1).

Bacteria belonging to phylum *Proteobacteria* were ubiquitous and the most abundant bacterial phylum across all the samples. A relatively higher abundance of this phylum was observed across all sediment samples S3 (60.06%), S1 (53.52%), and S2 (31.26%) as compared to the corresponding water samples, amongst which, the highest was W1 (34.72%), followed by considerably lower abundances in samples W3 (15%) and W2 (9.1%) (Figure 1A). Similarly, bacterial members of the phylum *Firmicutes* occurred at higher relative abundances in sediment samples (in S1 29.9%; S2 18.79%; and S3 16%) than in the water sample W1 (13.6%) (Figure 1A). In contrast, *Actinobacteria*, Candidate division TM7 and *Cyanobacteria* were more abundant in water samples, compared to the sediment samples (Figure 1A). Members of the *Actinobacteria* dominated the water samples (up to 80% in W3 sample) with a maximum of 4.5% detected in the sediment (sample S1). Likewise *Cyanobacteria* and Candidate division TM7 were more abundant in water samples at 30% (in sample W1) and 18% (in sample W3), in contrast, the sediment samples had 0.01% (S1 sample) and 0.01% (S2 sample), respectively. Certain bacterial groups including *Chloroflexi, Acidobacteria, Deinococcus, Fibrobacteres, Gemmatimonadetes, Nitrospirae*, and *Spirochaetes* were exclusive to sediment samples (Figure 1A). Within *Proteobacteria, Alpha*-, *Beta*-, *Gamma*-, and *Epsilonproteobacteria* were observed in all samples. Notably, members of *Gammaproteobacteria* were the most abundant (21–47%) amongst the three sediment samples and water sample W1 and formed a minor (1.4–3.1%) proportion of the remaining water samples (W2 and W3). *Gammaproteobacteria* were followed by *Alphaproteobacteria* in terms of relative abundance in sediment samples S1 (19.32%), S3 (11.8%), and water sample W1 (13.4%) (Figure 1A). In contrast, *Beta*-, *Delta*-, and *Epsilonproteobacteria* were present across the samples in relatively lower abundance (<1%). Amongst the uncultured Candidate divisions, members of Candidate division TM7 were the most abundant in the majority of the samples followed by members of Candidate divisions WS3, OP9, BRC1, and OD1 (Figure 1A). In summary, as compared to water samples, phyla *Proteobacteria* and *Firmicutes* were found to be significantly more abundant (*p* = 0.05) in sediment samples, whereas *Actinobacteria* followed by Candidate division TM7 and *Cyanobacteria* dominated the water samples of Lonar Lake (Figure 1B).

**Figure 2:**
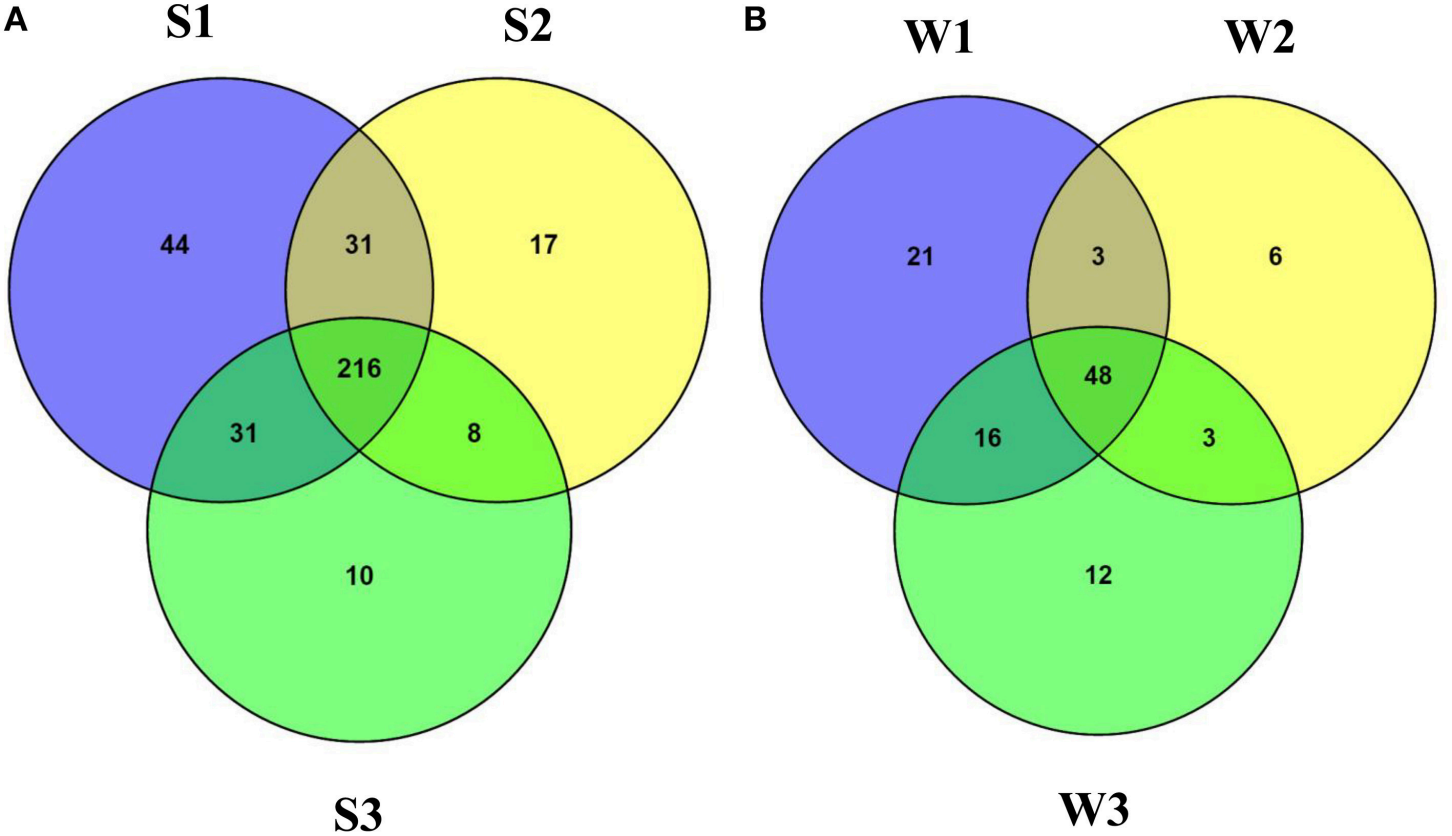
**Venn diagram demonstrating unique and shared OTUs at family level (A) among the sediment samples and (B) among the water samples of the Lonar Lake**.

**Figure 3:**
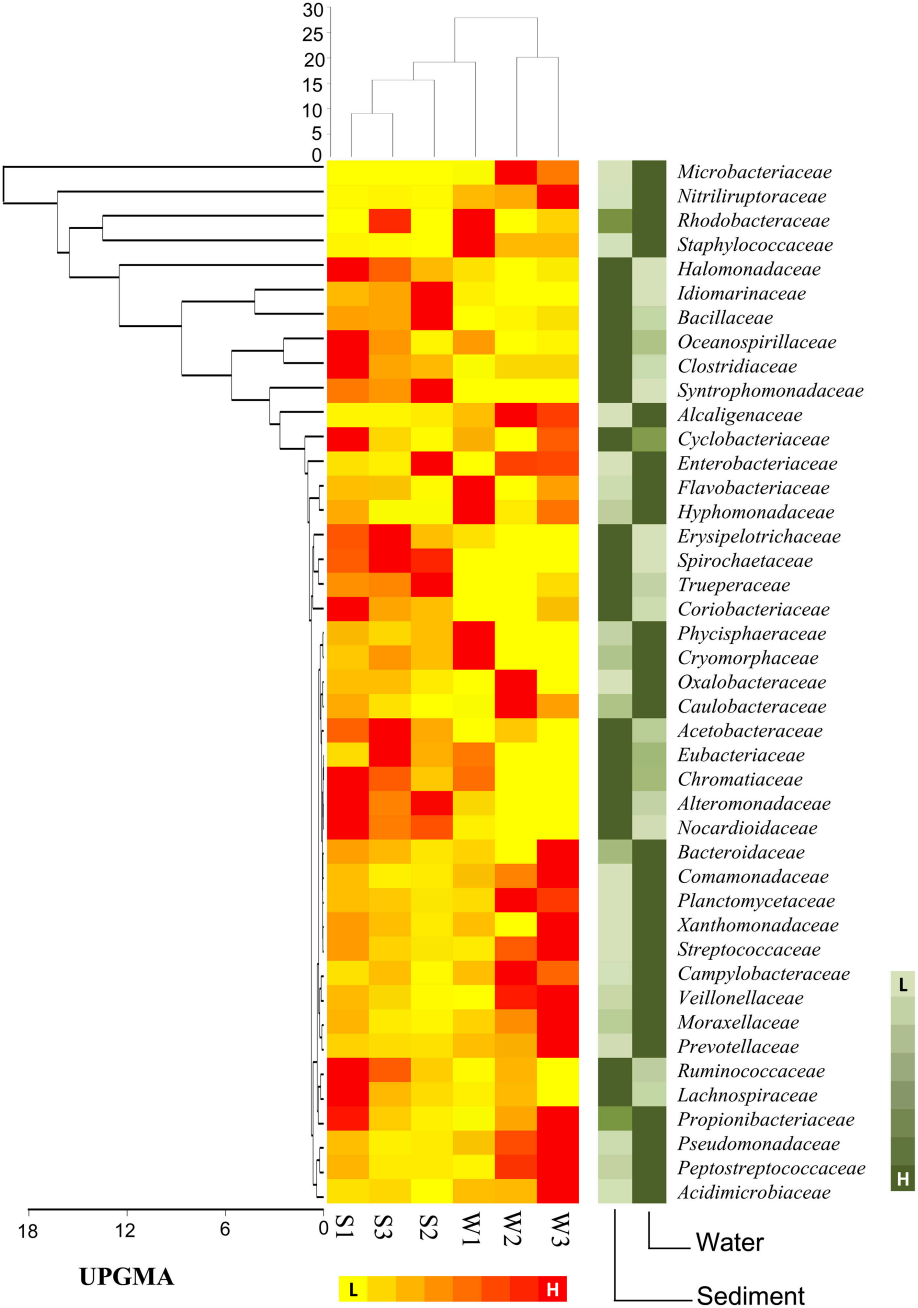
**Heat map shows distribution of major bacterial group (detected five or more than five samples) at family level**.

At the family level of taxonomic classification, a total of 216 common OTUs were observed in all the sediment samples with sample S1 containing the highest number of (44) unique OTUs (Figure 2A). Interestingly, water sample W1 (corresponding to sediment sample S1) also contained a higher number of unique OTUs (21) compared to the remaining water samples (Figure 2B). Only 159 OTUs could be assigned to the family level in our analysis. Among these, 21 OTUs were detected in all samples at ≥0.5% abundance and they were assigned to *Acidimicrobiaceae, Microbacteriaceae, Nitriliruptoraceae* (*Actinobacteria*), *Bacillaceae, Staphylococcaceae, Clostridiaceae, Peptostreptococcaceae* (*Firmicutes*), *Hyphomonadaceae, Rhodobacteraceae* (*Alphaproteobacteria*), *Alcaligenaceae* (*Betaproteobacteria*), *Enterobacteriaceae, Pseudomonadaceae* (*Gammaproteobacteria*) (Figure 3). Additionally, sediment samples had a higher relative abundance of *Halomonadaceae, Oceanospirillaceae, Clostridiaceae*, and *Syntrophomonadaceae*, whereas *Streptococcaceae, Caulobacteraceae*, and *Planctomycetaceae* predominated the water samples (Figure 3 and Figure S1). Members of the *Verrucomicrobiaceae, Methylococcaceae, Piscirickettsiaceae*, and *Methylobacteriaceae* were detected infrequently, occurring in four or fewer samples (Table S3). To understand the relatedness between samples analyzed, the Lonar Lake (sediment and water) samples were subjected to UPGMA at the family level of taxonomic distribution. Dendrogram analysis revealed that sediment samples shared high degree of relatedness as compared to their relatedness with the corresponding water samples (Figure 3).

### Statistical analyses

**Figure 4:**
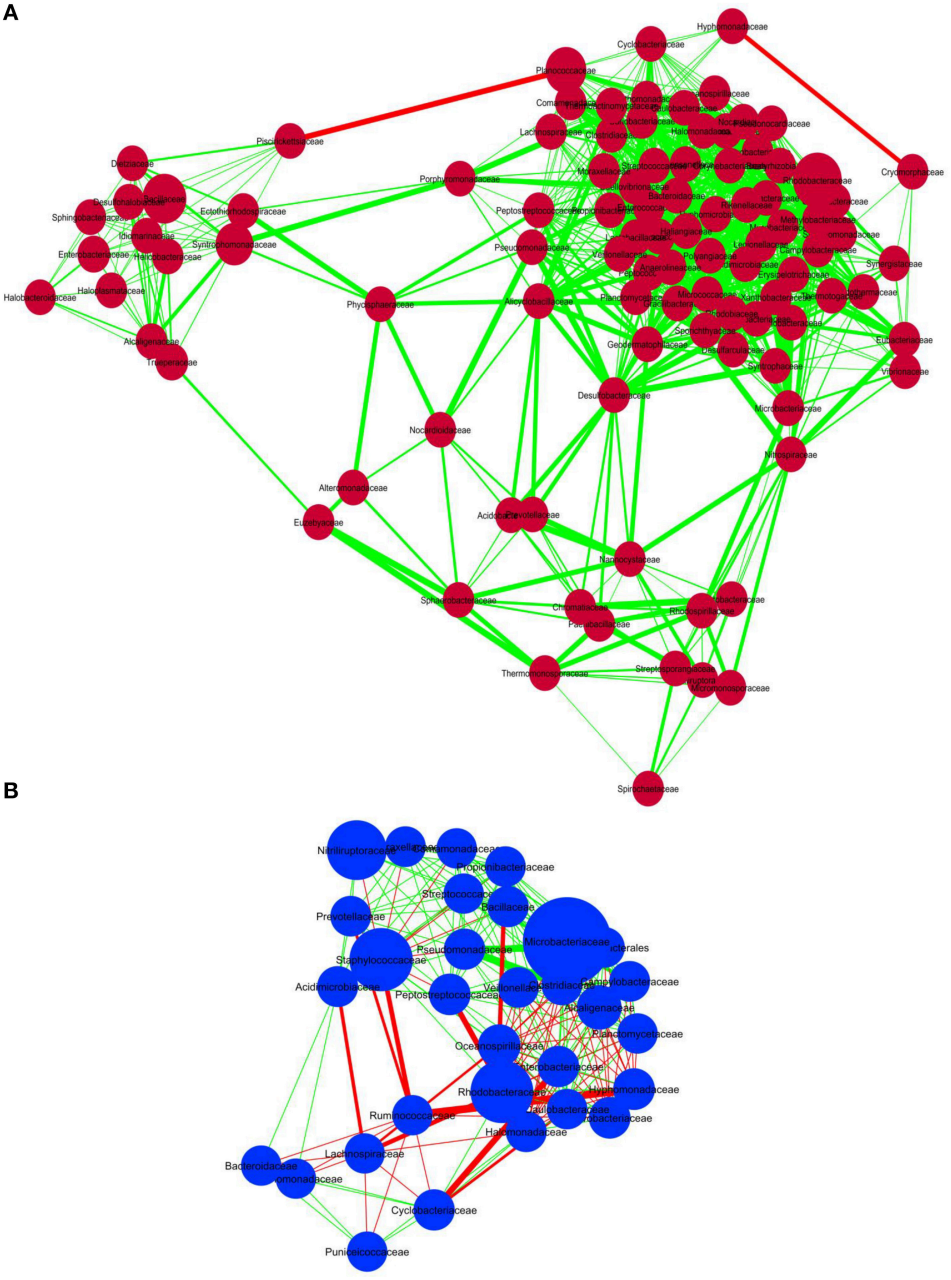
**Co-occurrence analysis of the bacteria members (A) sediment samples and (B) water samples of Lonar Lake at family level**. Diagram shows the interactions among different bacteria. Red line indicates negative association and green line indicates positive association. The thickness of the line indicates strength of interaction and the size of the bubble indicates the relative abundance of bacterial members.

In order to discern interactions between different bacterial groups at the family level, we applied co-occurrence analysis to the sequence dataset. We were able to detect 1117 and 219 interactions in sediment and water samples, respectively. Over 99% of the bacteria in the sediment samples and 66% of the bacteria in the water samples correlated positively with each other (Figure 4), whereas 34% of the bacterial groups in the water samples correlated negatively. Co-occurrence analysis also indicated the co-presence of *Phycosphaeraceae, Nocardioidaceae, Alteromonadaceae, Syntrophomonadaceae*, and *Peptostreptococcaceae* in the lake sediment (Figure 4A). Furthermore, the bacterial families *Desulfobacteraceae, Acidobacteraceae*, and *Alicyclobacillaceae* were found to correlate positively with each other, suggesting a core anaerobic population involved in biogeochemical cycling of carbon and sulfur. In contrast, negative correlations were observed for the families *Piscirickettsiaceae* with *Hyphomonadaceae*, and *Cryomorphaceae* with *Planococcaceae*, in the sediment environment. In water samples, the families *Pseudomonadaceae, Microbacteriaceae*, and *Clostridiaceae* correlated positively with each other, while the *Ruminococcaceae* correlated negatively with *Staphylococcaceae, Oceanospiraceae*, and *Lachnospiraceae* (Figure 4B).

**Figure 5:**
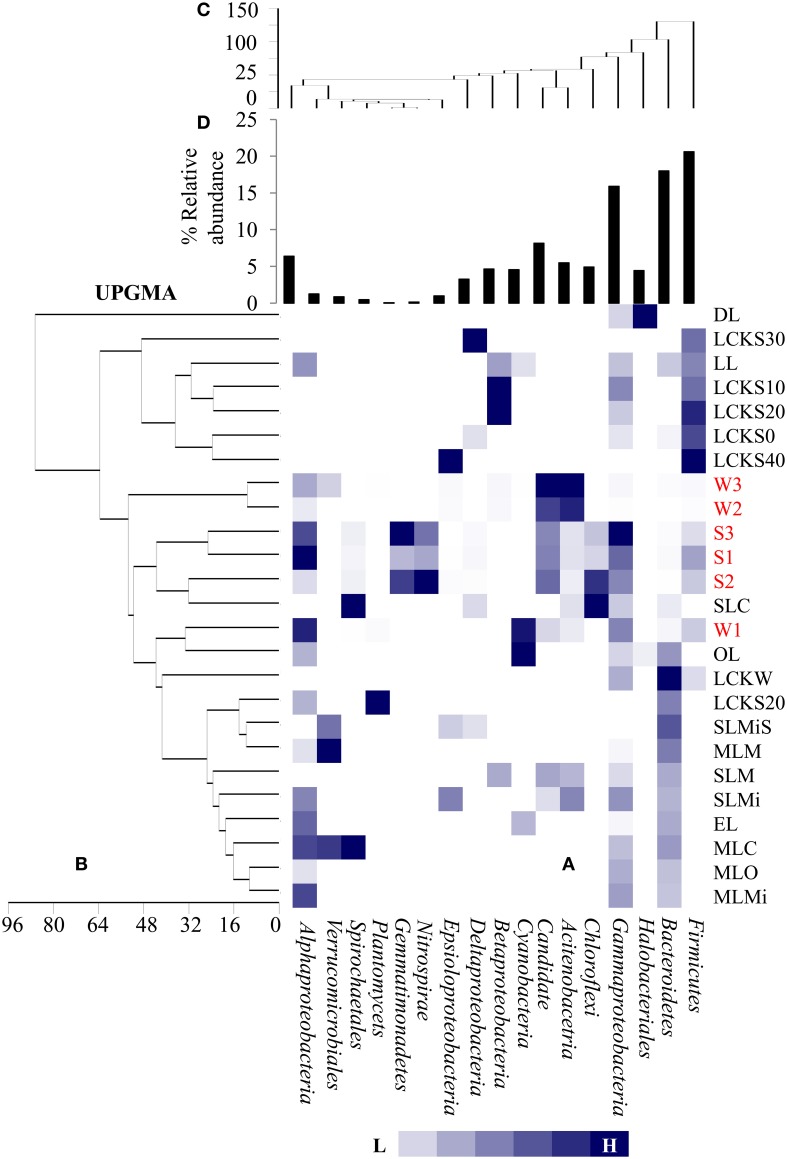
**Comparative analysis of bacterial community composition (A) among the present samples as well as those reported earlier from diverse hypersaline lake ecosystem, (B) UPGMA cluster analysis among the samples based on abundance of different bacterial groups as detected in different lake system, (C) UPGMA cluster analysis among the bacterial groups detected in diverse lake system and (D) relative abundance of the bacterial phylum**. The samples are denoted as follows: DL, Deep Lake (Colorado); LCKS30, Lake Chaka (China); LL, Lonar Lake (India); LCKS10, Lake Chaka (China); LCKS20, Lake Chaka (China); LCKS0, Lake Chaka (China); LCKS40, Lake Chaka (China); W3, Lonar Lake water (present study); W2, Lonar Lake water (present study); S3, Lonar Lake sediment (present study); S1, Lonar Lake sediment (present study); S2, Lonar Lake sediment (present study); SLC, Soap Lake Chemocline (Washington); W1, Lonar Lake water (present study); OL, Organic Lake (Eastern Antarctica); LCKW, Lake Chaka (China); LCKS20, Lake Chaka (China); SLMiS, Soap Lake Mixolimnion sediment (Washington); MLM, Mono Lake Monimolimnion (California); SLM, Soap Lake Monimolimnion (Washington); SLMi, Soap Lake Mixolimnion (Washington); EL, Echo Lake (Colorado); MLC, Mono Lake Chemocline (California); MLO, Mono Lake Oxycline (California); MLMi, Mono Lake Mixolimnion (California). The details of these samples are provided as Supplementary Information (Table S2).

For identifying relationships among the samples analyzed here and among previously reported soda lake samples with respect to bacterial community composition, a UPGMA based statistical analysis was performed. The soda lakes considered for this analysis were: Mono Lake, California; Chaka Lake, China; Ekho Lake, Colorado; Organic Lake, Eastern Antarctica; Deep Lake, Colorado; and Meromictic Soda Lake, Washington State. Previously published data combined with our data were used to compile a heat map based on relative abundance of individual phyla/class identified within the samples (Figure 5A). The comparison revealed that a number of taxonomic groups (*Gammaproteobacteria, Bacteroidetes, Firmicutes, Alphaproteobacteria*, and *Betaproteobacteria*) were observed consistently in geographically distinct hypersaline soda lakes. The analysis also demonstrated that other bacterial lineages (especially *Actinobacteria*, and Candidate divisions TM7) were conspicuous in Lonar Lake samples and Meromictic Soda Lake of Washington State samples, but not in other hypersaline soda lakes. The UPGMA based dendrogram indicated a strong relationship within Lonar Lake samples at or above a 50% similarity level (Figure 5B). Also, sample W1 (this study) was highly related to a sample from the Organic Lake (OL). Surprisingly, we observed a lack of relationship between results of our microbial census of Lonar Lake samples to a previously reported Lonar Lake sediment sample (LL). This unexpected finding could result from distinct time points of sample collection, seasonal variation, or differences in sample processing methodology. The Lonar Lake samples that were subjected to analysis in this study were dominated by *Proteobacteria, Actinobacteria, Firmicutes*, and *Cyanobacteria* whereas previous phylogenetic census revealed the dominance of *Proteobacteria* followed by *Bacteroidetes, Planctomycetes*, and *Actinobacteria* (Wani et al., 2006).

A second UPGMA was performed to analyze the relationship among the different bacterial groups present within the samples (Figure 5C). Evidently, *Alpha*-, *Beta*-, and *Gammaproteobacteria, Acidobacteria*, and *Bacteroidetes* were closely related across all the samples at 60% similarity level, suggesting their co-occurrence in a number of the hypersaline lake samples. Bacterial members of phyla *Firmicutes, Bacteroidetes*, and *Gammaproteobacteria* were abundant in a large proportion of studied soda lakes of the world including Lonar Lake (Figure 5D). Therefore, such analyses were not only able to estimate bacterial community composition among the diverse soda lake samples, but also identified bacterial phyla that might be favored in all hypersaline lakes. The abundance of these common bacterial groups in soda lakes clearly indicates their involvement in key aspects of a soda lake ecosystem.

## Discussion

This culture-independent molecular survey provides new insights into structure and composition of indigenous microbial communities within a meteorite impact hypersaline Lonar Lake ecosystem. The majority of bacterial members found in the samples belong to *Proteobacteria, Actinobacteria, Firmicutes*, and Candidate division TM7. Members of these groups were previously identified and reported in tropical soda lake ecosystems such as Lake Nakuru and Lake Bogoria too in the African Rift Valley as well as from the Lonar Lake ecosystem, where they transform methane, sulfur, elemental nitrogen or nitrate in autotrophic and/or chemotrophic metabolism under aerobic as well as anaerobic conditions (Duckworth et al., 1996; Jones and Grant, 1999; Chandrasekhar et al., 2002; Vargas et al., 2004; Wani et al., 2006). Physicochemical analyses of sediment and water samples demonstrated that Lonar Lake has a comparable characteristic of hypersaline lake ecosystem, based primarily on pH, salinity, TOC, and abundance of ${\text{CO}}_{3}^{2-}$ (Chandrasekhar et al., 2002; Wani et al., 2006; Joshi et al., 2008; Antony et al., 2010). Despite the existence of extreme alkaline and saline conditions in this lake, a diverse bacterial community was detected in the present study.

The sediment and water samples were distinct in terms of bacterial community composition. As indicated in previous studies, the bacterial community of the lake water in this study was also dominated by members of the *Actinobacteria*, Candidate division TM7 and WS3, and *Cyanobacteria* and sediment samples by *Firmicutes* and *Proteobacteria* (Chandrasekhar et al., 2002; Wani et al., 2006). Further, bacterial co-occurrence analysis showed a closer mutual association of bacterial community members in sediment samples, compared to water samples, suggesting competition for available resources being higher in the aquatic environment as compared to sediments (Faust et al., 2012). Syntrophic interactions between families like *Synotrophomonadaceae* in close associations with acetogenic families of *Clostridiaceae* were indicated by co-occurrence analysis, suggesting putative tightly controlled metabolite exchange in an extreme oligotrophic environment (Sieber et al., 2012). The chemical and physical limits of the habitat result in the development of co-existence or co-occurrence patterns within residing communities (Costello et al., 2009; Ofiteru et al., 2010; Langenheder and Székely, 2011). Possibly the higher abundance of different minerals (quartz, calcite, feldspars, etc.), halite salt, and reduced substances in sediments of Lonar Lake compared to water samples act as an abiotic control on microbial community composition (Wani et al., 2006; Antony et al., 2013). However, it could also be inferred that higher concentration of minerals and salts could also provide a suitable environment for the growth of many chemoheterotrophic bacteria. Therefore, the distinct patterns of bacterial community assembly across sediment and water are likely to be caused by differences in salinity, redox state and nutrient availability in this lake ecosystem.

Being a hypersaline environment, Lonar lake has a metabolically diverse halotolerant bacterial population. Within *Proteobacteria*, members of the *Halomonadaceae, Oceanospirillaceae*, and *Pseudomonadaceae* were prevalent in our taxonomic survey and have also been independently confirmed in such ecosystems using culture-based methods (Chandrasekhar et al., 2002; Wani et al., 2006; Joshi et al., 2008). These bacteria are known for their ubiquity and metabolic flexibility which includes their ability to tolerate extreme and/or oligotrophic environments, utilize diverse carbon compounds, and to maintain aerobic and anaerobic lifestyles (Brodie et al., 2006; Janssen, 2006; Wani et al., 2006). Additionally, *Firmicutes* were detected as a major group in Lonar Lake samples. Members of the *Bacillaceae, Planococcaceae*, and *Staphylococcaceae* were previously reported in various tropical alkaline lakes (Lake Nakuru, Lake Bogoria, and Lonar Lake) and were also observed in this study (Duckworth et al., 1996; Chandrasekhar et al., 2002; Vargas et al., 2004). They include members which are alkaliphiles, methylamine, methanol and dimethylsulfide utilizers, and have potential for metal resistance (Dijkhuizen et al., 1988; Anesti et al., 2005; Antony et al., 2010). Within Candidate divisions, members of Candidate division TM7 were found predominantly in water samples. Previously, the TM7 phylum was detected in a range of chemically and geographically diverse habitats, including groundwater, freshwater, seawater and deep-sea sediments (Hugenholtz et al., 2001; Di Rienzi et al., 2013; Basak et al., 2015; McLaughlin et al., 2015). Along with Candidate division TM7, the presence of other Candidate divisions namely WS3, OP9, and BRC1 in Lonar Lake may indicate a significant role for these groups in this lake ecosystem.

Lonar Lake sediment microbiota plays an important role in the utilization of C1 compounds. Previous investigations have suggested methanotrophy (Antony et al., 2010, 2013). A number of Proteobacterial families such as *Methylococcaceae, Piscirickettsiaceae* (*Gammaproteobacteria*), and *Methylobacteriaceae* (*Alphaproteobacteria*) were detected during this investigation and have been biochemically implicated in cycling biogenic methane in sediments, which prevents its escape into the atmosphere, by oxidizing and assimilating methane-derived C1 compounds (Whalen et al., 1990; Hanson and Hanson, 1996; Antony et al., 2010). Sulfur-metabolizing bacteria, such as members of the *Ectothiorhodospiraceae, Peptococcaceae, Desulfohalobiaceae*, and *Desulfonatronaceae*, were also found in Lonar Lake as minor populations. These bacteria were previously identified as chemophototrophs (*Ectothiorhospiraceae*), or chemoheterotrophs (*Peptococcaceae, Desulfohalobiaceae*, and *Desulfonatronaceae*) which could utilize various inorganic sulfur compounds as electron donors or electron acceptors (Sorokin et al., 2006, 2011; Foti et al., 2007). Bacterial members of the family *Ectothiorhodospiraceae* are particularly remarkable in their ability to grow in saturated alkaline environments whereas *Peptococcaceae, Desulfohalobiaceae*, and *Desulfonatronaceae* are well known sulfate-reducing bacteria (Zhilina et al., 1997; Robertson et al., 2001; Pikuta et al., 2003). Hence, the co-occurrence of sulfur-oxidizing and sulfate-reducing bacteria in soda lakes suggests the existence of a functionally linked intercycle coupling, i.e., redox junctions between C and S cycles. The sediments of hypersaline lakes contain a high level of a number of reduced substances, including inorganic compounds like sulfide or ammonia. Owing to the accumulation of such reduced substances at the bottom of the lake compared to water, the water column of hypersaline lakes may be deprived of essential nutrients (Jellison et al., 1996; Antony et al., 2010, 2013). In agreement with earlier studies, sediments showed a higher C:N ratio along with increased ammonium, sulfate and phosphate concentrations compared to water samples; in turn, these results suggest increased nutrient turnover/mineralization and higher productivity in sediments of the lake. Hence, it is apparent that the geochemical gradients in Lonar Lake sediment and water samples contribute to the overall microbial structure in the lake, and also result in higher bacterial diversity/OTUs in Lonar Lake sediments, compared to the water column, thus bearing a clear influence on the activities of the indigenous microbial communities of the lake ecosystem.

## Conclusions

We present here the first study to integrate a uniform framework of Lonar Lake sediment and water ecosystems from a microbiological aspect. Detailed 16S rRNA gene sequencing using high-throughput Ion torrent platform revealed a diverse and distinct landscape of bacterial fauna in both sediments and water samples with prevalence of the phyla *Gammaproteobacteria, Alphaproteobacteria, Actinobacteria, Firmicutes*, and Candidate divisions TM7 and WS3. Bacterial taxa of distinct phylogenetic clusters carry out central biogeochemical functions such as methylotrophy and sulfate reduction, and suggest complex biogeochemical interactions in this ecosystem that await specific functional characterization by targeted gene expression and geochemical studies.

## Author contributions

Conceived and designed the experiments: DP, SK, SM, SC, SS, NM, YS. Performed the experiments: SK, SM, SC, SS, NM, DP. Analyzed the data: DP, SB. Contributed reagents/materials/analysis tools: YS. Manuscript preparation: DP. Manuscript improvement: SC, DP, SK, SB, NM, SS.

### Conflict of interest statement

The authors declare that the research was conducted in the absence of any commercial or financial relationships that could be construed as a potential conflict of interest.

## Supplementary material

Supplementary Figure1 **Significant differences of bacterial abundance at family level as a result of a Fisher exact test between the sediment and water samples conducted with the STAMP program**. Bacterial abundance in the sediment sample has a positive difference between proportions (blue circles), whereas bacterial abundance in the water sample has a negative difference between proportions (red circles). Bars on the left represent the proportion of each bacterial family abundance in the samples. Bacterial abundance difference with a *p*-value of >0.05 were considered to be significant.

Supplementary Table1 **Details of the primers use**.

Supplementary Table2 **Details of the samples used for comparing microbial composition among other samples (Figure 5)**.

Supplementary Table3 **Bacterial family detect in less number of samples (≤4 samples) of Lonar Lake**.
