# Supplementary figures and images for: Exploration of Microbial Diversity and Community Structure of Lonar Lake: The Only Hypersaline Meteorite Crater Lake within Basalt Rock
Source: Front Microbiol. 2016 Jan 22;6:1553. doi: 10.3389/fmicb.2015.01553 (PMC4722114; doi:10.3389/fmicb.2015.01553)

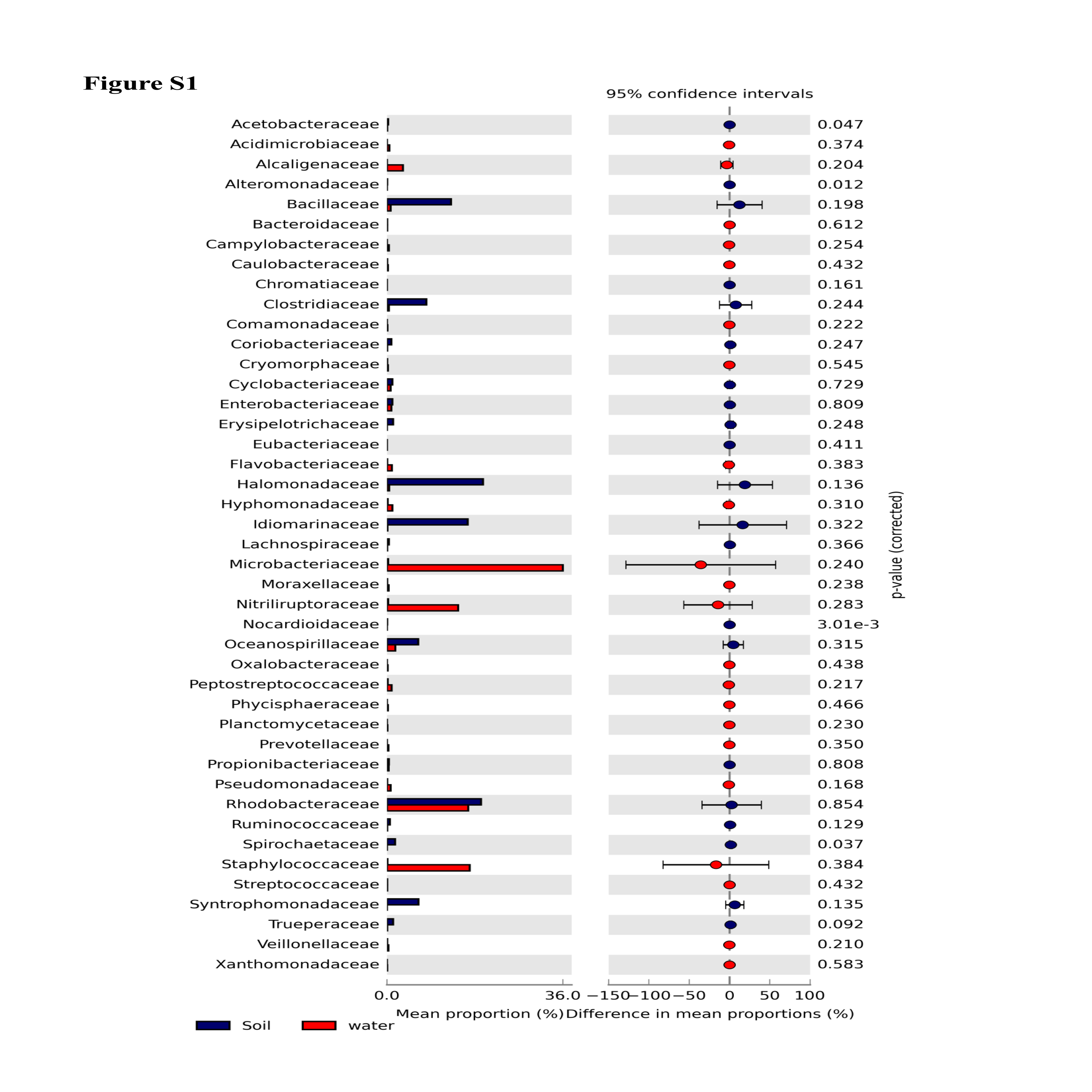

Supplement: Supplementary Figure1 — Significant differences of bacterial abundance at family level as a result of a Fisher exact test between the sediment and water samples conducted with the STAMP program. Bacterial abundance in the sediment sample has a positive difference between proportions (blue circles), whereas bacterial abundance in the water sample has a negative difference between proportions (red circles). Bars on the left represent the proportion of each bacterial family abundance in the samples. Bacterial abundance difference with a p-value of >0.05 were considered to be significant. [file Image1.TIF]
