# Supplementary material for: Exploration of Microbial Diversity and Community Structure of Lonar Lake: The Only Hypersaline Meteorite Crater Lake within Basalt Rock
Source: Front Microbiol. 2016 Jan 22;6:1553. doi: 10.3389/fmicb.2015.01553 (PMC4722114; doi:10.3389/fmicb.2015.01553)
Supplement: Supplementary Table1 — Details of the primers use. [file Table1.DOCX]

| **Sample Code** | **A** | **Barcode** | **Adapter** | **Primer (5'-3')** |
| --- | --- | --- | --- | --- |
| **S1** | CCATCTCATCCCTGCGTGTCTCCGACTCAG | TCTGGATGAC | GAT | ACTCCTACGGGAGGCAGCAG |
| **S2** | CCATCTCATCCCTGCGTGTCTCCGACTCAG | TCTAGAGGTC | GAT | ACTCCTACGGGAGGCAGCAG |
| **S3** | CCATCTCATCCCTGCGTGTCTCCGACTCAG | AAGAGGATTC | GAT | ACTCCTACGGGAGGCAGCAG |
| **W1** | CCATCTCATCCCTGCGTGTCTCCGACTCAG | CTGACCGAAC | GAT | ACTCCTACGGGAGGCAGCAG |
| **W2** | CCATCTCATCCCTGCGTGTCTCCGACTCAG | TCTAACGGAC | GAT | ACTCCTACGGGAGGCAGCAG |
| **W3** | CCATCTCATCCCTGCGTGTCTCCGACTCAG | TCCTCGAATC | GAT | ACTCCTACGGGAGGCAGCAG |
|  | p1 |  |  |  |
| **Reverse primer** | CCTCTCTATGGGCAGTCGGTGAT |  |  | TTACCGCGGCTGCTGGCAC |

**Table S1** Details of the primers use
