# Supplementary material for: Exploration of Microbial Diversity and Community Structure of Lonar Lake: The Only Hypersaline Meteorite Crater Lake within Basalt Rock
Source: Front Microbiol. 2016 Jan 22;6:1553. doi: 10.3389/fmicb.2015.01553 (PMC4722114; doi:10.3389/fmicb.2015.01553)
Supplement: Supplementary Table2 — Details of the samples used for comparing microbial composition among other samples (Figure 5). [file Table2.DOCX]

**Table S2** Details of the samples used for comparing microbial composition among other samples (Figure 7)

| **Sl No.** | **Sample Id** | **Sample description** | **References** |
| --- | --- | --- | --- |
| 1 | DL | Sediment sample, Deep Lake, Colorado | Bowman et al. FEMS microbiology letters, 183(1), 81-88:2000. |
| 2 | LCKS30 | Sediment sample, Lake Chaka, China | Jiang et al. Applied and environmental microbiology, 72(6), 3832-3845:2006. |
| 3 | LL | Water sample, Lonar Lake, India | Wani et al. Research in microbiology, 157(10), 928-937:2006. |
| 4 | LCKS10 | Sediment sample, Lake Chaka, China | Jiang et al. Applied and environmental microbiology, 72(6), 3832-3845:2006. |
| 5 | LCKS20 | Sediment sample, Lake Chaka, China | Jiang et al. Applied and environmental microbiology, 72(6), 3832-3845:2006. |
| 6 | LCKS0 | Sediment sample, Lake Chaka, China | Jiang et al. Applied and environmental microbiology, 72(6), 3832-3845:2006. |
| 7 | LCKS40 | Sediment sample, Lake Chaka, China | Jiang et al. Applied and environmental microbiology, 72(6), 3832-3845:2006. |
| 8 | SLC | Water sample, Soap Lake Chemocline, Washington | Dimitriu et al. Applied and environmental microbiology, 74(15), 4877-4888:2008. |
| 9 | OL | Sediment sample, Organic Lake, Eastern Antarctica | Bowman et al. FEMS microbiology letters, 183(1), 81-88:2000. |
| 10 | LCKW | Water sample, Lake Chaka, China | Jiang et al. Applied and environmental microbiology, 72(6), 3832-3845:2006. |
| 11 | LCKS20 | Sediment sample, Lake Chaka, China | Jiang et al. Applied and environmental microbiology, 72(6), 3832-3845:2006. |
| 12 | SLMiS | Sediment sample, Soap Lake Mixolimnion, Washington | Dimitriu et al. Applied and environmental microbiology, 74(15), 4877-4888:2008. |
| 13 | MLM | Water sample, Mono Lake Monimolimnion California | Humayoun et al. Applied and environmental microbiology, 69(2), 1030-1042:2003. |
| 14 | SLM | Water sample, Soap Lake Monimolimnion, Washington | Dimitriu et al. Applied and environmental microbiology, 74(15), 4877-4888:2008. |
| 15 | SLMi | Sediment sample, Soap Lake Mixolimnion, Washington | Dimitriu et al. Applied and environmental microbiology, 74(15), 4877-4888:2008. |
| 16 | EL | Sediment sample, Echo Lake, Colorado | Bowman et al. FEMS microbiology letters, 183(1), 81-88:2000. |
| 17 | MLC | Water sample, Mono Lake Chemocline, California | Humayoun et al. Applied and environmental microbiology, 69(2), 1030-1042:2003. |
| 18 | MLO | Water sample, Mono Lake Oxycline, California | Humayoun et al. Applied and environmental microbiology, 69(2), 1030-1042:2003. |
| 19 | MLMi | Water sample, Mono Lake Mixolimnion, California | Humayoun et al. Applied and environmental microbiology, 69(2), 1030-1042:2003. |
